# Editorial: Emerging roles of the gut microbiota in the pathogenesis of metabolic disorders, volume II

**DOI:** 10.3389/fendo.2024.1415705

**Published:** 2024-05-27

**Authors:** Jieying Liu

**Affiliations:** Center for Biomarker Discovery and Validation, National Infrastructures for Translational Medicine (PUMCH), Institute of Clinical Medicine, Peking Union Medical College Hospital, Peking Union Medical College, Chinese Academy of Medical Sciences, Beijing, China

**Keywords:** gut microbiota, metabolic disorders, microbial metabolites, biomarkers, metabolism

The global surge in metabolic disorders, encompassing obesity, type 2 diabetes mellitus (T2DM), metabolic dysfunction-associated fatty liver disease (MAFLD), cardiovascular diseases, and a spectrum of cancers, presents a profound and urgent public health challenge. This escalation underscores the critical need to deepen our understanding of metabolic physiology and to delineate further biomarkers and targets for the prevention of metabolic disorders. The origins of metabolic disorders are multifaceted, involving both inherited genetic susceptibilities and a range of acquired factors throughout an individual’s life. While inherited metabolic disorders (IMDs) typically constitute a collection of uncommon genetic anomalies (1), the majority of metabolic disorders are precipitated by exogenous influences, including environmental factors and lifestyle choices (2).

Central to this discussion is the intestinal microbiome, significantly influenced by external variables, notably diet and physical activity. The gut microbiome plays pivotal roles in various essential physiological processes that underpin metabolic equilibrium, such as nutrient digestion, metabolite production, and immune system modulation. Consequently, the dynamic interplay between gut microbiota and the host could illuminate the mechanisms through which external factors disrupt metabolic homeostasis.

This Research Topic delves into the critical role of the gut microbiota in influencing metabolic disorders. Such exploration is poised to shed light on the complexities underlying metabolic disorders, paving the way for the development of innovative diagnostic, prevention and treatment strategies. By focusing on the gut microbiota’s impact, this collection of studies contributes valuable insights into potential mechanisms driving metabolic dysregulation and highlights novel biomarkers and intervention targets.

A substantial volume of research has been directed toward identifying personalized diagnostic biomarkers for metabolic disorders. Neri-Rosario et al. investigated the gut microbiota profiles (16S rRNA gene sequencing) of 410 Mexican patients, who were categorized as normoglycemic, prediabetic, or with type 2 diabetes (T2D). They identified specific microbial taxa that could predict T2D presence in comparison to normoglycemic individuals through a machine learning framework. Furthermore, this research team (Esquivel-Hernández et al.) conducted a re-analysis of 16S rRNA gene amplicon data to examine the gut microbiome dynamics across various T2D stages within a Mexican cohort. They discovered a progressive dissociation among bacterial associations correlating with disease advancement and proposed a set of genera implicated in T2D progression. These findings underscore the heightened sensitivity of the microbial community to disturbances in T2D, offering a set of microbial genera as potential biomarkers for distinguishing between healthy controls and T2D patients, as well as among different T2D progression stages.

Two reviews have elucidated the pivotal roles of gut microbiota and their metabolites in the pathogenesis of diabetic nephropathy (DN) (Zhao et al.) and thyroid disorders (Mendoza-León et al.). Zhao et al. have detailed the specific changes in the gut microbiome associated with DN, highlighting the potential roles of microbial metabolites—such as short-chain fatty acids, bile acids, trimethylamine-N-oxide, uremic toxins, and hydrogen sulfide—in the disease process. Furthermore, they summarized preclinical animal studies on gut microbiota intervention strategies for DN. Thyroid dysfunctions, categorized into hypothyroidism and hyperthyroidism, exhibit uncertain causality. Mendoza-León et al. concentrated on the impact of gut microbiota on thyroid disorders, articulating the intricate interplay between nutrition, thyroid functionality, and gut microbiota. They particularly underscored the influence of short-chain fatty acids on thyroid health. Additionally, Zhang et al. reported on gut microbiota imbalances and reduced propionic acid levels in patients with Cushing’s disease, characterized by chronic hypercortisolism. These contributions offer fresh insights into the gut microbiota’s impact on metabolic disorders, extending beyond the extensively documented conditions of T2D and obesity.

Animal studies have been instrumental in elucidating the link between alterations in gut microbiota and changes in the functionality of target organs. Lee et al. demonstrated that sleep deprivation coupled with a high-fat diet may disrupt the brain’s inflammatory system to induce obesity, potentially through mechanisms mediated by inosine-5’ phosphate that influence microbiota-gut-brain interactions. Li and Shi utilized a model of chronic intermittent hypoxia in rats to probe the pathogenesis of sleep apnea syndrome. This study revealed significant modifications in serum pro-inflammatory cytokines, gut microbiota composition, serum and palate tissue metabolites, and cAMP-related protein expression, providing a comprehensive view of the condition’s multifaceted nature. These findings advocate for the implementation of further causative experiments to substantiate these preliminary conclusions, promising to enhance our understanding of the complex interplay between gut microbiota and systemic physiological responses.

This Research Topic extensively explores the alterations in gut microbiota and their metabolites and their pivotal role in the progression of metabolic disorders. It aims to elucidate the regulatory impact of gut microbiota on the functionality of target organs. Additionally, the potential diagnostic value of specific gut microbiota configurations and strategies for gut microbiome intervention are critically examined, marking a significant stride towards the translation of this research into clinical applications. Despite these advancements, the need for rigorous cause-effect validation and expanded clinical research remains pressing. Collectively, these studies underscore the integral role of gut microbiota in the etiology of metabolic disorders, suggesting a promising avenue for novel therapeutic interventions.

## Author contributions

JL: Writing – original draft, Writing – review & editing.
